# The Gut Microbial Signature of Gestational Diabetes Mellitus and the Association With Diet Intervention

**DOI:** 10.3389/fcimb.2021.800865

**Published:** 2022-01-14

**Authors:** Na Wu, Jingwei Zhou, Heng Mo, Qing Mu, Huiting Su, Mei Li, Yimeng Yu, Aiyu Liu, Qi Zhang, Jun Xu, Weidong Yu, Peng Liu, Guoli Liu

**Affiliations:** ^1^ Department of Central Laboratory & Institute of Clinical Molecular Biology, Peking University People’s Hospital, Beijing, China; ^2^ Department of Gynecology and Obstetrics, Peking University People’s Hospital, Beijing, China; ^3^ Department of Stomatology, Peking University People’s Hospital, Beijing, China; ^4^ Department of Clinical Nutrition, Peking University People’s Hospital, Beijing, China

**Keywords:** GDM, gut microbiota, diet intervention, SCFAS-producing genera, *Firmicutes/Bacteroidetes* ratio

## Abstract

Gestational diabetes mellitus (GDM) is a high-risk pregnancy complication that is associated with metabolic disorder phenotypes, such as abnormal blood glucose and obesity. The link between microbiota and diet management contributes to metabolic homeostasis in GDM. Therefore, it is crucial to understand the structure of the gut microbiota in GDM and to explore the effect of dietary management on the microbiota structure. In this study, we analyzed the composition of the gut microbiota between 27 GDM and 30 healthy subjects at two time points using Illumina HiSeq 2500 platform. The taxonomy analyses suggested that the overall bacteria clustered by diabetes status, rather than diet intervention. Of particular interest, the phylum *Acidobacteria* in GDM was significantly increased, and positively correlated with blood glucose levels. Moreover, Partial least-squares discriminant analysis (PLS-DA) revealed that certain genera in the phyla *Firmicutes, Bacteroidetes, Proteobacteria*, and *Lentisphaerae* characterized the GDM gut microbiota. Correlation analysis indicated that blood glucose levels and BMI index were correlated with the relative abundance of SCFAS-producing genera. Through the comparison between the GDM and healthy samples with or without diet intervention, we discovered that the role of short-term diet management in GDM processes is associated with the change in the *Firmicutes/Bacteroidetes* ratio and some specific taxa, rather than an alternative gut microbial pattern. Our study have important implications for understanding the beneficial effects of diet intervention on the specific gut microbiota and thus possibly their metabolism in pregnant women with GDM.

## Introduction

The intestinal microbiota is a robust ecosystem inhabited by nearly 100 trillion bacteria (Savage, 1977). In recent years, extensive attention has been given to the gut microbiota during pregnancy. Over the course of a healthy pregnancy, the body undergoes substantial hormonal, immunological, and metabolic changes (Newbern and Freemark, 2011; Koren et al., 2012). In predisposed women, these physiological changes may lead to the development of gestational diabetes mellitus (GDM). GDM is defined as abnormal glucose regulation with onset or first recognition during pregnancy and is one of the most common complications during pregnancy, with an incidence of 2–6% of all pregnancies (Cani and Delzenne, 2007; Vijay-Kumar et al., 2010). The clinical incidence of GDM in China is currently presenting a dramatic increasing trend and the prevalence of GDM was up to 19.7% among 15,194 pregnant women in 15 hospitals in Beijing by 2013 (Zhu et al., 2017; Juan and Yang, 2020). In the context of nonpregnant obesity, recent work suggests a role for gut microbiota in driving metabolic diseases, including diabetes, weight gain, and reduced insulin sensitivity (Turnbaugh et al., 2006; Cani and Delzenne, 2007; Vijay-Kumar et al., 2010; Scheithauer et al., 2020). Researchers understand that the intestinal flora has an important function in the development of GDM with the notions relating the intestinal flora to metabolic disease (Koren et al., 2012; Crusell et al., 2018; Wang et al., 2018). Several studies revealed the role of gut microbiota in GDM. Gut dysbiosis was observed in GDM, the gut microbial taxa at phylum, family and genus levels were characterized by change of abnormal bacterial composition, which was associated with higher blood glucose (Kuang et al., 2017; Liu et al., 2019). GDM is a transient state, and GDM patients are commonly treated by diet management to keep blood glucose within the normal range and reduce the risk of GDM complications (Buchanan et al., 2012). However, very few data from observational studies are available about whether diet interventions performed on GDM patients affect the community structure of the gut microbiota. Diet, particularly long-term eating habits, is known to be one of the drivers of microbiota variation (Bassis, 2019; Johnson et al., 2019). Recent clinical studies have shown the importance of routine dietary recommendations for GDM patients, showing a better microbial pattern at the end of the study (Ferrocino et al., 2018). However, the comparison between healthy pregnant women and individuals with GDM under routine dietary management remains uncertain.

In this study, we characterized the different patterns of the gut microbiota between GDM and healthy pregnancies in the second trimester of pregnancy. Then, comparison of microbial structure between healthy pregnant women and individuals with GDM under routine dietary management were assessed, to evaluate the role of short-term diet management on GDM gut microbiota. The aim of the present study was to provide an update on the existing knowledge of the specific structure of the gut microbiota in Chinese GDM women and to elucidate the influence of diet management on the GDM gut microbiota.

## Material and Methods

### Patient Recruitment

This study was approved by the Conjoint Health Research Ethics Board of Peking University People’s Hospital, and informed consent forms were signed by all of the subjects prior to participation in this study. All experiments were performed in accordance with the approved guidelines and regulations.

Diagnosis of GDM is based on the results of the fasting 75 g oral glucose tolerance test (OGTT) at 24–28 weeks gestation. One or more elevated level(s) is sufficient for a diagnosis of GDM. The threshold values of OGTT (5.1 at 0 hour, 10.0 at 1 hour and 8.5 at 2 hours during OGTT) are based on the diagnostic criteria recommended by the International Association of the Diabetes and Pregnancy Study Groups in 2011. Twenty-seven GDM were recruited based on the criteria.

Thirty healthy subjects were selected based on matched age and pregnancy period, no complicating diseases and no antibiotic use during the 3-month period prior to sample collection. All subjects who met the following criteria were excluded: complicating diseases (such as known diabetes mellitus, hypertension, cardiovascular, pulmonary, autoimmune, joint, liver or kidney diseases; thyroid dysfunction; or any other disease), prebiotics/probiotics use, and antibiotic use during pregnancy.

The prepregnancy weight was self-reported; weight and height were measured at the time of enrollment. BMI was calculated as weight divided by the square of height. Arterial blood pressure (BP) was measured from the left arm with the participant in a sitting position after at least 10 min of rest with a mercury sphygmomanometer with the appropriate cuff size. The measurements for BP were taken by trained medical personnel at enrollment.

**Figure 1:**
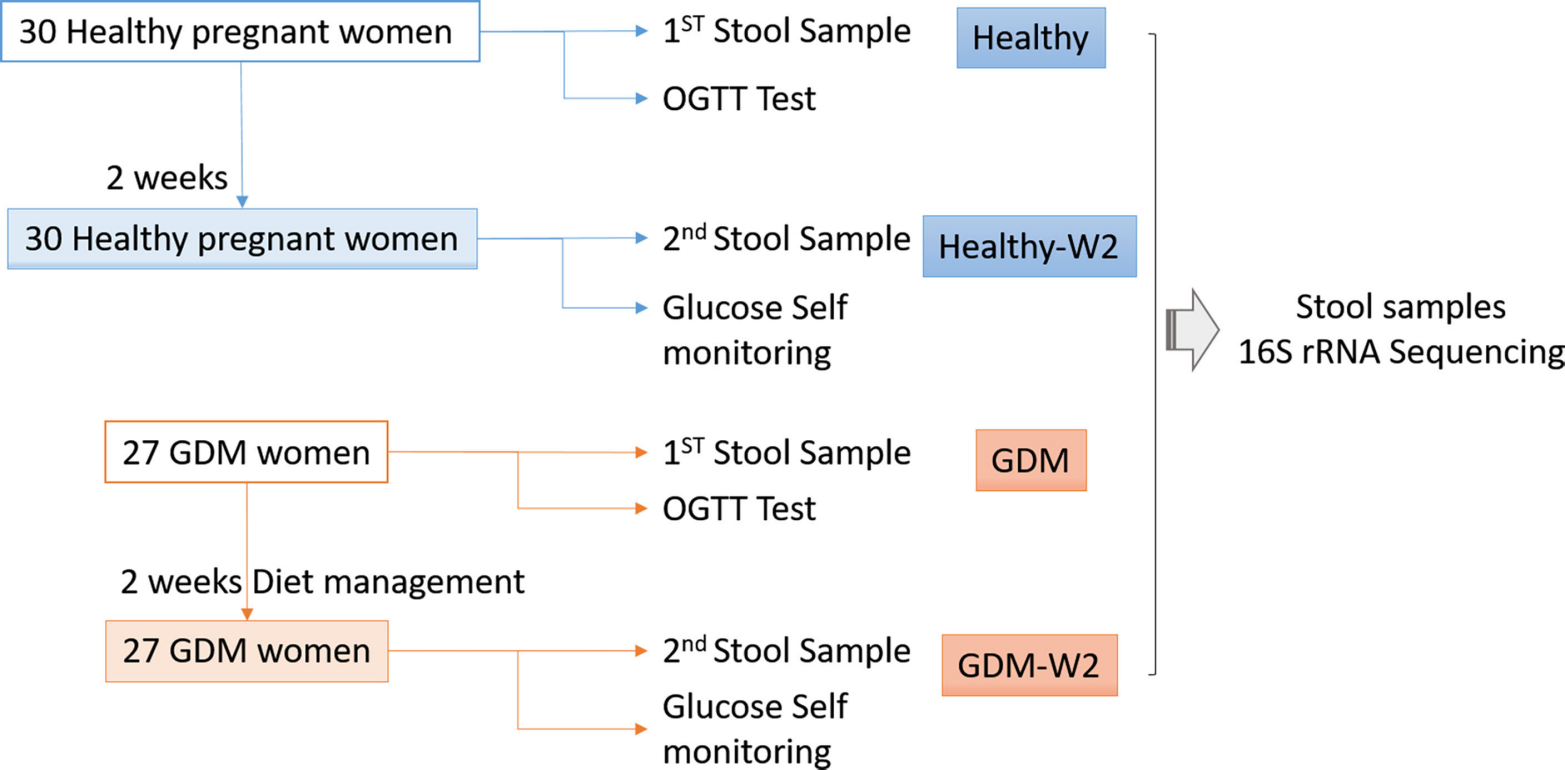
Flow chart illustrating the recruitment of GDM and healthy subjects.

A flow chart illustrating the recruitment strategy of GDM and healthy subjects is shown in **Figure 1**. Clinical data from 27 GDM patients and 30 healthy controls are shown in **Table 1**. All 27 GDM patients and 30 healthy pregnant women were from the Peking University People’s Hospital. The mean age of the subjects was 32.7 ± 3.3 years for the GDM group and 31.4 ± 2.9 years for the healthy group. There were no differences in age or nulliparity rate between the two groups. The pregnancy BMI value of the GDM group was 24.2 ± 4.4, which was significantly higher than the value of 21.4 ± 2.8 of the healthy group (*P*=0.0059), and the same trend was observed for the BMI at enrollment (27.1 ± 4.3 vs. 25.0 ± 2.9, GDM vs. healthy, *P*=0.038). The GDM group had a markedly higher systolic BP (SBP) value than that of the control group (mean 125.3 ± 11.8 vs. 115.8 ± 14.2, GDM vs. healthy, *P*=0.008), and an increased diastolic BP (DBP) value was found in GDM women compared to that of healthy women (mean 78.8 ± 9.5 vs. 73.6 ± 8.8, GDM vs. healthy, *P*=0.038). In the OGTT test, the GDM group had higher values at 0 h, 1 h and 2 h than the values of the healthy group (all *P*<0.001).

**Table 1:** The clinical characteristics of all the GDM patients differ from those of the healthy participants.

	GDM(Mean ± SD)	Healthy(Mean ± SD)	*P* value
Number	27	30	
Age	32.7 ± 3.3	31.4 ± 2.9	0.11
Prepregnancy weight (kg)	63.5 ± 12.2	57.3 ± 8.9	0.031
BMI (kg/m^2^)	24.2 ± 4.4	21.4 ± 2.8	0.0059
Enrollment weight (kg)	71.1 ± 12.4	66.9 ± 9.5	0.15
BMI (kg/m^2^)	27.1 ± 4.3	25.0 ± 2.9	0.038
Nulliparous (number)	22/27	24/30	
Systolic BP (mmHg)	125.3 ± 11.8	115.8 ± 14.2	0.008
Diastolic BP (mmHg)	78.8 ± 9.5	73.6 ± 8.8	0.038
OGTT (mmol/L)			
0 min	5.2 ± 1.4	4.3 ± 0.3	0.001
60 min	10.1 ± 1.6	7.3 ± 1.4	<0.0001
120 min	8.8 ± 1.3	6.4 ± 1.2	<0.0001

### Diet Management for the GDM Women

The initial treatment of GDM involves diet modification, glucose monitoring, and moderate exercise (Blumer et al., 2013; American Diabetes Association, 2014). All the GDM participants in the study received 2 weeks of dietary management and nutritional recommendations at enrollment, which showed the guidelines for the GDM subjects. Participants were considered as adhering to the given dietary recommendations based on the wide consumption of cereals, legumes, skimmed dairy products, low-fat meat and fish, vegetables, fruit and no consumption of processed baked goods, fast foods, soft drinks, juices and alcohol. The diet had a target composition of 35%-45% carbohydrates (80% complex carbohydrates with a low glycaemic index and 20% simple carbohydrates), 18%-20% protein (50% animal and 50% vegetable) and 35% fat (16% mono-unsaturated, 10% polyunsaturated and 9% saturated) with moderately low saturated fat levels, fiber intake of at least 20–25 g/day. The daily recommended calories were divided into small frequent meals to avoid ketonuria and acidosis, which frequently occurs because of prolonged fasting. The nutritionist was in continuous contact with the enrolled GDM subjects, through weekly telephone contact, to remain updated regarding the nutritional condition of the subjects as the study progressed. Patients were instructed to self-monitor their blood glucose by finger-prick capillary blood glucose tests at least 4 times per day.To reduce the effect of prebiotics/probiotics use on the composition of the gut microbiota, general suggestion were imposed on the healthy participants, including no peppery food and no yogurt intake.

### Stool Sample Collection and DNA Extraction

After providing written informed consent, all subjects were contacted for detailed instructions on how to collect and transport the stool sample. Stool samples of 57 subjects were collected at the time of enrollment for the first time. The second stool samples for GDM subjects were collected at the end of the study after the 2-week dietary intervention. For healthy pregnant women, the second stool samples were collected at the end of 2 weeks without dietary management intervention. Stool samples were self-collected by all the participants using the specimen collection kit as instructed. The fecal samples were collected at home, transferred to the hospital and immediately stored at −80°C until DNA extraction. DNA was extracted from stool samples using the QIAamp DNA Stool Mini kit protocol (Qiagen, Germany). During the stool collection, one GDM sample at enrollment from one patient (G28) were limited, and the second sample was collected the other day, which changed the serial number to G28-2 at enrollment and G28-3 at the end of study.

### Library Generation

The V4 region of the 16S rRNA gene was amplified using 515F (5’-GTGCCAGCMGCCGCGGTAA -3′) and 806R (5’-GGACTACHVGGGTWTCTAAT -3’). The V4-specific primer regions were associated with the barcode and linker primers (**Supplementary Table 1**). The amplicon library from each sample was prepared by TruSeq^®^ DNA PCR-Free Sample Preparation Kit.Then the 250-bp nucleotide paired-end sequencing was performed using the HiSeq 2500 genome analyzer (Illumina HiSeq 2500, USA).

### 16S rRNA Amplicon Sequence Analysis

The RDP Classifier was used to assign all of the 16S rRNA gene sequences to a taxonomic hierarchy. The assembled reads were analyzed. The relative abundances of the various phyla, families and genera in each sample were computed and compared between the GDM patients and the healthy subjects. The trimmed reads were clustered into operational taxonomic units (OTUs) at 97% identity. Then, taxonomic assignment of the OTUs was determined based on a GreenGene classifier. The gut microbiota from 114 fecal samples was profiled using high throughput 16S rRNA gene sequencing of the V4 variable region. A total of 6,315,267 high quality combined sequences (55,397 ± 10,783 sequences per sample) were ultimately produced. And 3,271 OTUs were identified and functionally labeled using QIIME1 platform.

The comparison of the bacterial α-diversity of these samples was performed using the Chao1 richness index, ACE index, Shannon’s diversity index and observed species. The reads displaying greater than 0.1% abundance in both groups were further analyzed *via* partial least-squares discriminant analysis (PLS-DA) to visualize the differences between two groups using the standard Simca-p1 software (version 12.0; http://www.umetrics.com/). The Principal Co-ordinates Analysis (PcoA) analyzed were performed based on Unweighted Unifrac distance metric, and Adonis test was further used to reveal the significance of microbiota composition changes between the groups (the separation of clusters).

Phylogenetic Investigation of Communities by Reconstruction of Unobserved States (PICRUSt) was performed to preidict the functional differences of gut microbiota between GDM samples and healthy samples based on Kyoto Encyclopedia of Genes and Genomes (KEGG).

### Statistical Analysis

The microbial comparisons between the GDM and healthy groups were performed using the Mann-Whitney test. Associations between clinical indices and gut microbiota were evaluated by the Spearman rank correlation coefficient method. The difference in alpha-diversity between groups during GDM and non-GDM was assessed using Student’s t test. Statistical analysis of the clinical data was performed using SPSS (Statistical Package for Social Sciences) 22.0 software (SPSS Inc., Chicago, IL, USA). *P*<0.05 was considered significantly different.

## Results

### Differences in Fecal Microbial Communities Between the Healthy and GDM Groups

**Figure 2:**
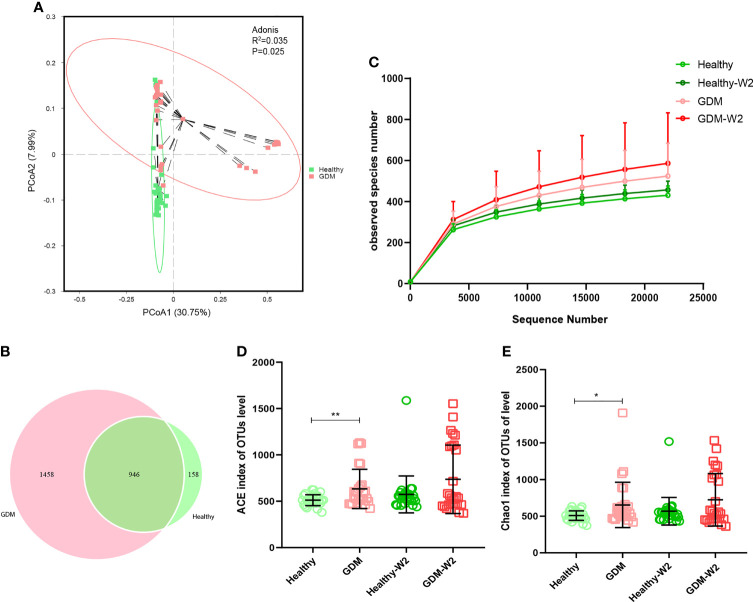
Comparison of the fecal microbiota composition between the GDM and healthy groups. **(A)** Principal coordinate analysis (PCoA) at the OTU level between the GDM and healthy groups at enrollment. **(B)** Venn diagram illustrating the overlap of the OTUs identified in the fecal microbiota between the GDM and healthy groups at enrollment. **(C)** Observed species of 4 groups, including the GDM and healthy and the GDM-W2 and healthy-W2 groups. D & **(E)** Alpha-diversity based on the ACE index and Chao 1 index at the OTU level. Mann-Whitney test, GDM vs. healthy, **P<0.01, *P<0.05.

To demonstrate the GDM microbiota signature, we explored the microbial composition of pregnant women with GDM. First, we performed PCoA using OTU relative abundance, and we observed discrete clustering of intestinal microbiota in the GDM and healthy groups at enrollment (**Figure 2A**). Additionally, shared or unique OTUs in the GDM and control groups were assessed to detect whether GDM has an effect on the gut microbiota. We found that the GDM group had more unique OTUs than the control group, with approximately 60.6% (1458/2404) unique OTUs compared with 14.3% (158/1104) in healthy women, signifying that GDM patients largely harbor unique inhabitant niches (**Figure 2B**).

The observed species of GDM samples were higher than healthy samples (**Figure 2C**). The ACE and Chao1 indices for alpha-diversity were both significantly increased in the GDM at enrollemnt (**Figures 2D, E**), suggesting increased commensal diversity in GDM patients compared with healthy sujects. Increasing trend of alpha-diversity was also observed between the Healthy-W2 and GDM-W2 (diet management) groups, suggesting that the microbial pattern of women with GDM is distinct from that of healthy subjects at enrollment and at the end of the study. In addition, the microbial diversity analysis was performed by means of Shannon index, there was no difference among the healthy and GDM women at enrollment and at the end of study (**Supplementary Figure 1**).

### Microbiota Structure of GDM Patients Based on Taxonomic Comparison

**Figure 3:**
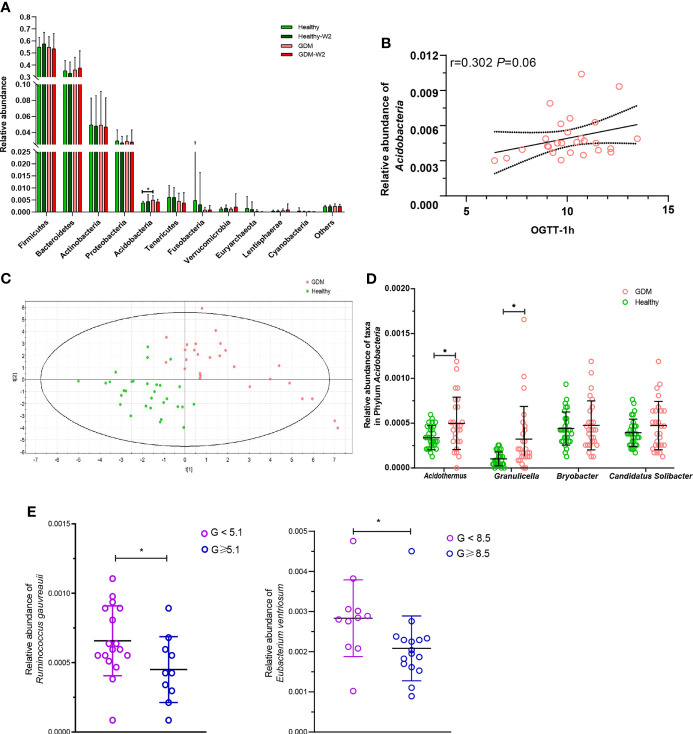
Abundances of taxa in GDM and healthy participants. **(A)** Comparison of the relative abundances at the phylum level among the four GDM and non-GDM groups. The Mann–Whitney test was used to evaluate the two groups. *P<0.05. **(B)** PLS-DA score plots based on the relative abundances of microbiota between the GDM and healthy groups. **(C)** Correlation between the relative abundance of the phylum *Acidobacteria* and the 1-h OGTT measurement. Spearman analysis, R=0.302, *P*=0.06. **(D)** Comparison of the relative abundances of *Acidothermus, Granulicella, Bryobacter*, and *Candidatus_Solibacter* in the phylum *Acidobacteria* in the GDM and healthy groups. Mann-Whitney test, GDM vs. control, *P < 0.05. **(E)** The relative abundances of *Ruminococcus gauvreauii* and *Eubacterium ventriosum* were highly correlated with the OGTT values at 0 h and 2 h. Mann-Whitney test, GDM vs. healthy, *P < 0.05.

To further demonstrate these variations corresponding to the structure of the gut microbiota in GDM, we compared the bacterial abundance between groups at the phylum level (**Figure 3A**). No significant differences were observed between the healthy subjects and the GDM subjects at enrollment for most of the phyla, with the exception of *Acidobacteria*, which was found to be 0.51% in the GDM group compared with 0.37% in the healthy group (*P*=0.001).

The microbial compositions at the phylum level for each sample at enrollment and at the end of the study are shown in **Supplementary Figure 2**. Interestingly, *Acidobacteria* was associated with increased levels of blood glucose in the 0-h OGTT (**Figure 3B**).

Next, we compared taxa at the genus level. The PLS-DA method was performed (**Figure 3C**). Forty-nine key genera with variable importance in projection (VIP) scores >1 were identified that differentiated the GDM and healthy groups (**Table 2**). We then clustered the samples according to the relative abundance of the 49 genera. Twenty-seven genera were enriched in the GDM microbiota samples, with 4 genera (*Acidothermus, Granulicella, Bryobacter*, and *Candidatus_Solibacter*) belonging to the phylum *Acidobacteria*. Among them, *Acidothermus* and *Granulicella* were significantly enriched in the GDM group (**Figure 3D**). Seven genera belonging to *Proteobacteria*, including *Citrobacter*, *Burkholderia*, *Acidibacter*, and *Bilophila*, were significantly increased in the GDM intestinal microbiota (*P*<0.05). The genera *Eubacterium*, *Holdemania*, and *Tyzzerella*, in the phylum *Firmicutes*, were rarely detected in women with healthy pregnancy microbiota compared with women with GDM. The remaining 22 genera of the 49 key phylotypes were higher in healthy pregnant microbiota, some of which even disappeared in GDM patients. One genus, *Ruminococcaceae_UCG-010*, belonging to *Firmicutes*, was highly enriched in the healthy group. Additionally, *Akkermansia* (*P*=0.067) and *Coprococcus_2* (*P*=0.027) were increased in healthy subjects. *Akkermansia* was recently proven to be a crucial player in maintaining the integrity of the gastrointestinal tract. In nonpregnant adults with metabolic syndrome and type 2 diabetes, *Akkermansia* is reported to be depleted as well (Everard et al., 2013; Hills et al., 2019; Macchione et al., 2019). Our findings suggest that the gut microbiota of women with GDM has similarities with the microbiota reported in patients with type 2 diabetes and associated intermediary metabolic traits. At the OTU level, a reduced abundance of *Akkermansia* has previously been reported in the third trimester of healthy pregnant women (Yao et al., 2020).

**Table 2:** Forty-nine key genera with VIP >1 in the GDM and healthy groups.

Genus with VIP ≥1	GDM mean	Healthy mean	GDM/Healthy	*P* value	Phylum
*Citrobacter*	0.000316	2.41E-05	up	0.048	*Proteobacteria*
*Bradyrhizobium*	0.000422	8.64E-05	up	0.065	*Proteobacteria*
*Eubacterium*	0.00012	3.4E-05	up	0.001	*Firmicutes*
*Granulicella*	0.000323	0.000102	up	0.001	*Acidobacteria*
*Holdemania*	0.000187	7.5E-05	up	0.014	*Firmicutes*
*Succinivibrio*	8.81E-05	3.54E-05	up	0.212	*Proteobacteria*
*Oscillibacter*	0.000211	9.06E-05	up	0.44	*Firmicutes*
*Tyzzerella*	0.000856	0.000368	up	0.007	*Firmicutes*
*Holdemanella*	0.009481	0.004176	up	0.162	*Firmicutes*
*Paraprevotella*	0.000994	0.000578	up	0.126	*Bacteroidetes*
*Victivallis*	0.00056	0.000344	up	0.042	*Lentisphaerae*
*Desulfovibrio*	0.000458	0.00029	up	0.479	*Proteobacteria*
*Lachnospiraceae*	0.002291	0.001517	up	0.137	*Firmicutes*
*Burkholderia*	0.000824	0.000551	up	0.027	*Proteobacteria*
*Acidothermus*	0.000499	0.000338	up	0.034	*Acidobacteria*
*Acidibacter*	0.000677	0.000508	up	0.405	*Proteobacteria*
*Mucilaginibacter*	0.00037	0.00028	up	0.02	*Bacteroidetes*
*Candidatus_Solibacter*	0.000474	0.000394	up	0.404	*Acidobacteria*
*Ruminiclostridium_9*	0.001163	0.00098	up	0.141	*Firmicutes*
*Ruminococcus_gauvreauii*	0.000581	0.000491	up	0.214	*Firmicutes*
*unidentified_Ruminococcaceae*	0.001548	0.001359	up	0.949	*Firmicutes*
*Roseburia*	0.028429	0.025656	up	0.482	*Firmicutes*
*Bilophila*	0.002439	0.002216	up	0.179	*Proteobacteria*
*Alistipes*	0.011983	0.010959	up	0.354	*Bacteroidetes*
*Bryobacter*	0.000475	0.00044	up	0.968	*Acidobacteria*
*Odoribacter*	0.001495	0.001395	up	0.302	*Bacteroidetes*
*Dorea*	0.007676	0.007233	up	0.678	*Firmicutes*
*Lachnospiraceae_NK4A136*	0.002712	0.002715	down	0.438	*Firmicutes*
*Eubacterium_ruminantium*	0.00633	0.006883	down	0.26	*Firmicutes*
*Bifidobacterium*	0.033865	0.038103	down	0.56	*Acidobacteria*
*Ruminococcaceae_UCG-013*	0.001251	0.00142	down	0.994	*Firmicutes*
*Tyzzerella_3*	0.002118	0.002482	down	0.073	*Firmicutes*
*Ruminococcaceae_UCG-005*	0.00281	0.003448	down	0.452	*Firmicutes*
*Ruminococcaceae_UCG-002*	0.005792	0.00715	down	0.056	*Firmicutes*
*Ruminococcaceae_NK4A214*	0.00144	0.001797	down	0.083	*Firmicutes*
*Eubacterium_ventriosum*	0.00239	0.003046	down	0.207	*Firmicutes*
*Enterococcus*	0.001193	0.001627	down	0.09	*Firmicutes*
Megasphaera	0.001971	0.00306	down	0.749	*Firmicutes*
*Lachnospiraceae_UCG-003*	0.000269	0.000419	down	0.11	*Firmicutes*
*Coprococcus_2*	0.005271	0.008583	down	0.027	*Firmicutes*
*Ruminiclostridium_5*	0.001904	0.003111	down	0.009	*Firmicutes*
*Ruminococcaceae_UCG-010*	0.000848	0.001403	down	0	*Firmicutes*
*Sarcina*	0.000126	0.000252	down	0.001	*Firmicutes*
*Butyrivibrio*	0.000455	0.000927	down	0.02	*Firmicutes*
*Intestinimonas*	3.93E-05	8.64E-05	down	0.07	*Firmicutes*
*Akkermansia*	0.000189	0.000435	down	0.067	*Verrucomicrobia*
*Weissella*	7.87E-05	0.000217	down	0.002	*Firmicutes*
*Prevotella_2*	0.001153	0.003598	down	0.108	*Bacteroidetes*

To further examine the relationship between these VIP genera in GDM, we evaluated their abundance based on the results of the OGTT. The threshold values (5.1 at 0 h, 10.0 at 1 h and 8.5 at 2 h during the OGTT) are based on the diagnostic criteria recommended by the International Association of the Diabetes and Pregnancy Study Groups in 2011. As shown in **Figure 3E**, two short chain fatty acids producing and anti-inflammatory bacteria were highly correlated with the OGTT value at 0 h and 2 h. The relative abundance of *Ruminococcus gauvreauii* was observed depleted in GDM women with abnormal OGTT value at 0 h (*P*=0.046), and the relative abundance of *Eubacterium ventriosum* was decreased in GDM women with the abnormal OGTT value at 2 h (*P*=0.009, Mann-Whitney test).

### Microbiota Signature After Dietary Intervention

We found that GDM patients developed a microbial pattern with higher alpha-diversity after diet management (**Figures 2D, E**). Compared with the GDM samples, the GDM-W2 samples showed some distinct taxa with VIP scores >1, according to the PLS-DA analysis (**Supplementary Figure 3**).

**Figure 4:**
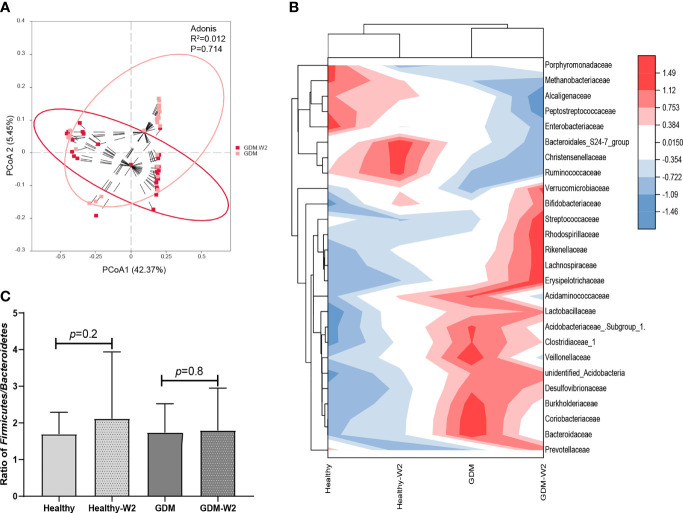
The microbial pattern after diet management. **(A)** Principal coordinate analysis (PCoA) at the OTU level between the GDM-W2 and healthy-W2 groups. **(B)** Heatmap analysis of the differential taxa at the family level. **(C)** Ratio of *Firmicutes/Bacteroidetes* among the GAM and non-GDM groups with or without diet intervention.

At the family level, GDM-W2 samples showed decreased pathogenic taxa (*Acidaminococcaceae*, *Enterobacteriaceae*, and *Bacteroidaceae*) and increased *Bifidobacteriaceae* and butyric acid-producing bacteria (*Prevotellaceae* and *Lachnospiraceae*) compared with the GDM microbial samples at enrollment, suggesting a better pattern driven by the 2 weeks of diet management. One more interesting observation is that bacterial lineages is more similar between all the cases at two time points, rather than diabetes status (**Figures 4A, B**), which is consistent with the findings shown in **Figure 2A**. It is presumed that the influence of maternal gestational diet on the phylogenetic structure of the intestinal microbiota during pregnancy remains underexplored in well-controlled models. To investigate whether the microbiota can be driven by dietary management for GDM in pregnancy, the two dominant groups of beneficial bacteria, *Bacteroidetes* and *Firmicutes*, were analyzed. At the phylum level, a slightly increase in the *Firmicutes*/*Bacteroidetes* (F/B) ratio in late pregnancy was exhibited in the GDM group compared with the non-GDM group (**Figure 4C**). Previous studies indicated that a higher *Firmicutes*/*Bacteroidetes* ratio was associated with obesity and other metabolic syndromes (Roselli et al., 2017; Magne et al., 2020), and an aggravation of low-grade inflammation (Ley et al., 2006). Here, we found that after 2 weeks of diet therapy, the relative abundance of *Bacteroidetes* in GDM samples showed an increased trend, and the abundance of *Firmicutes* decreased slightly (**Figure 3A**). More importantly, the ratio of *Firmicutes*/*Bacteroidetes* did not increase in GDM-W2 fecal samples compared with GDM samples at enrollment (*P*=0.8) (**Figure 4C**). However, an increased proportion of *Firmicutes*/*Bacteroidetes* (*P*=0.2) developed in healthy pregnancies (healthy-W2 samples).

Four genera (*Acidothermus, Granulicella, Bryobacter*, and *Candidatus_Solibacter*) belonging to the phylum *Acidobacteria* were increased in the GDM group, compared with healthy group

(**Figure 3D**). Furthermore, we evaluated the levels of the 4 genera in GDM with dietary management (**Supplementary Figure 4**). A total of 66.7% (18/27) of GDM subjects showed decreased levels of the genus *Acidothermus* after 2 weeks of diet management. In contrast, 59.3% (16/27) of GDM samples showed decreased levels of the genera *Granulicella, Bryobacter*, and *Candidatus Solibacter* after 2 weeks of diet management.

The gut microbiota assumes essential physiological functions in the host, which may influence the whole-body metabolism. Therefore, we investigated the effects of dietary intervention on the gut microbiota function by means of Picrust. There was no significant difference between Healthy and GDM groups before and after the diet intervention. Only high expression of amino acid metabolism and lipid metabolism were observed in the GDM samples at enrollment compared with healthy samples (**Supplementary Figure 5**), indicating GDM affected functional categories of gut microbiota.

### Association Between Fecal Microbiota and Clinical Parameters

**Figure 5:**
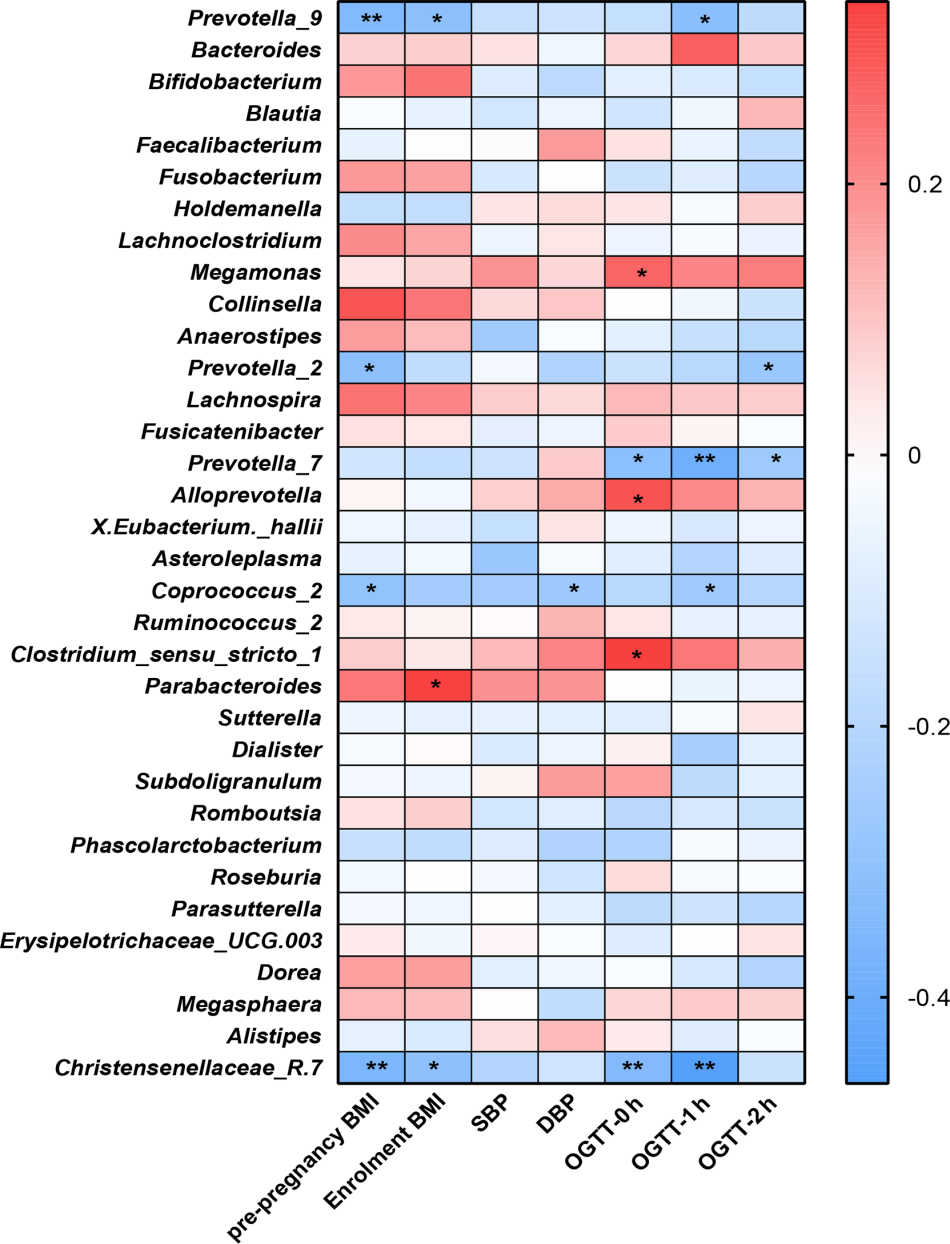
Heatmap analysis of the correlation between the gut microbiota composition and clinical scores. **P<0.01, *P<0.05.

We examined the correlations between the OGTT values (0 h, 1 h and 2 h), BMI indices (prepregnancy and at enrollment), blood pressure values (SBP and DBP) and the genera of the fecal microbiota (**Figure 5**).

The distribution of correlation coefficients by heatmap analysis showed that the *Coprococcus_2*, *Christensenellaceae*_R.7, and *Prevotella* groups (*Prevotella_2, Prevotella_7* and *Prevotella_9*) were negatively correlated with the OGTT value, BP values and BMI index (*P*<0.05); among them, *Coprococcus_2* was significantly increased in the healthy group compared with the GDM group.

*Parabacteroides* showed positive correlations with BMI at enrollment (*P*<0.05). Additionally, *Alloprevotella, Megamonas* and *Clostridium_sensu_stricto-1* showed positive correlations with GDM-correlated clinical measures and OGTT values at 0 h (*P*<0.05). Previous studies observed that the genus *Megamonas* was increased in GDM patients in late pregnancy.

## Discussion

Studies support a causal role for the gut microbiota in the development of type 2 diabetes, insulin resistance and obesity (Kreznar et al., 2017). In this study, we compared the composition of the human intestinal microbiota between GDM patients and healthy subjects using a culture-independent Illumina HiSeq 2500 platform. The aim of the present study was to identify gut microbiota dysbiosis in GDM subjects and the associated microbial changes in GDM-W2 samples after diet intervention for 2 weeks and compare them with the basal GDM microbial composition. We observed a marked shift in the microbiota composition at the phylum and genus levels in GDM samples compared with healthy samples and identified the microbial pattern of GDM-W2 samples after a 2-week dietary intervention.

Gut dysbiosis in women with GDM was mainly characterized by changes in microbiota diversity. It was previously reported that an increase was found in the alpha-diversity in the third trimester of GDM women when compared to the level of the control group (Cortez et al., 2019). Regarding alpha-diversity, we used the ACE and Chao1 indices and found significant separation in the alpha-diversity between GDM and non-GDM individuals at their enrollment and at the end of the study, indicating dysbiosis of the gut microbiota in GDM women compared with healthy pregnant women. To further identify gut microbial dynamics, the different bacterial taxa were compared within the GDM and non-GDM groups. At the phylum level, the abundance of *Acidobacteria* was significantly greater in the gut microbiota of GDM samples and was associated with increased levels of blood glucose in the 0-h OGTT (**Figure 3B**). In particular, we observed significant elevation of *Acidothermus* and *Granulicella* belonging to the phylum *Acidobacteria* in the GDM group. The phylum *Acidobacteria* was reported in the gut microbiome of obese individuals (Nardelli et al., 2020) and was shown to contain a host of genes involved in diverse metabolic pathways, as evidenced by their pan-genomic profiles in the soil microbiota (Kalam et al., 2020). Further exploration of these genetic attributes and more in-depth insights into GDM mechanics and dynamics would lead to a better understanding of the functions and biological significance of this elevated phylum in the GDM gut environment.

Several bacterial groups at the genus level were detected to be different in the GDM and healthy groups, such as *Megamonas* assigned to the phylum *Firmicutes.* The relationships between gastrointestinal *Megamonas* and metabolic disorders such as obesity and type 2 diabetes have recently been discovered (Chen et al., 2020b). Differential abundance testing showed that *Megamonas, Bacteroides*, and *Eubacterium* were statistically associated with food addition (Dong et al., 2020). In addition, a recent study suggested that the abundance of *Megamonas*, which is closely related to childhood obesity, increased in the gut microbiota of obese children (Chen et al., 2020b). Elevated genera of *Megamonas* have also been reported to be associated with higher blood glucose at an individual level level (Kuang et al., 2017; Crusell et al., 2018; Cortez et al., 2019; Huang et al., 2021). Of particular interest, we revealed the association between gut *Megamonas* and GDM. Our results showed that *Megamonas* was positively correlated with higher blood glucose in the OGTT test at 0 h in the GDM samples at enrollment (**Figure 5**). Members of *Megamonas* are known to produce acetic and propionic acid, which is beneficial for the balance of glucose uptake (Chen et al., 2020a). Systemic disorders of glucose metabolism might be modulated by the related gut microbiota. Further study to explore the composition of *Megamonas* and the production of metabolites involved in glucose homeostasis *in vitro* and *in vivo* is very important.

Short-chain fatty acids (SCFAs), especially acetate, propionate and butyrate, are the end products of the intestinal microbial fermentation of dietary fibers and resistant starch. It is well documented that plasma and colonic SCFAs are associated with metabolic syndromes, i.e., obesity and type 2 diabetes (Hu et al., 2018). SCFAs, namely, acetate, butyrate, and propionate, have been reported to affect metabolic activities at the molecular level. Acetate affects the metabolic pathway through the G protein-coupled receptor (GPCR) and free fatty acid receptor 2 (FFAR2/GPR43). The FFAR2 signaling pathway regulates insulin-stimulated lipid accumulation in adipocytes and inflammation (He et al., 2020; Kumar et al., 2020). *Coprococcus_2*, an acetate-producing bacteria (Pryde et al., 2002; Huang et al., 2021), was found to be negatively correlated with the OGTT value at 1 h, BP values and prepregnancy BMI index (*P*<0.05) by Spearman analysis and was significantly higher in the healthy group than in the GDM group. *Coprococcus* was also proven to be altered in the fecal microbiota of patients with polycystic ovary syndrome, which is a metabolic disorder (Guo et al., 2021). Guo et al. (Guo et al., 2018; Pan et al., 2021) found that *Coprococcus* deletion is implicated in many of the outcomes, including glucose homeostasis. The importance of an association between the deletion of the *Coprococcus* genus and high levels of blood glucose at 1-h in the OGTT measure is therefore supported by the acetate-producing effect. Furthermore, other SCFA-producing taxa, including *Prevotella_2*, *Prevotella_7*, and *Prevotella_9*, were found to be negatively associated with OGTT measures and the BMI index separately, indicating a beneficial effect on blood glucose in GDM subjects (De Vadder et al., 2016). We presumed that acetate arising from *Coprococcus_2* members and succinate from *Prevotalla* members are important for energy metabolism and have a mainly protective role in relation to healthy pregnancy. Thus, the observed absence of the *Coprococcus_2* and *Prevotella* groups in the fecal microbiota of GDM could be a possible microbial driving force for GDM. A better understanding of the microbial ecology of colonic acetate- and succinate-producing bacteria, especially the *Coprococcus_2* and *Prevotella* groups, may help to explain the influence of diet on the acetate and succinate supply and may contribute to the development of new approaches for optimizing microbial activity for diet management for GDM subjects. *Eubacterium ventriosum*, another SCFAs producer, had been found negative correlated with visceral fat area (VFA) (Nie et al., 2020). Moraes et al. reported that the abundance of *E. ventriosum* were associated to better cardiometabolic profile (de Moraes et al., 2017). Consistent with our study, the data demonstrated a significant decrease of gut *Eubacterium ventriosum* from GDM subjects with abnormal OGTT values at 2 h (**Figure 3E**). Combined with these findings, we presumed that the expression of the SCFAs producers are critical for energy homeostasis during pregnancy. Further studies investigating the targets and signaling pathways of SCFAs in the GDM microbial, and the modulation of SCFAs-producing bacteria by diet intervention would benefit for GDM management.

Therefore, to further identify the role of diet intervention during GDM pregnancy, we analyzed the ratio of *Firmicutes/Bacteroidetes*, and a higher ratio was proposed as an eventual biomarker of obesity and other metabolic syndromes compared with normal-weight individuals (Magne et al., 2020). Our data showed different increases in the *Firmicutes/Bacteroidetes* ratio between the GDM and non-GDM groups. Healthy W2 samples without diet management showed a nearly significant increase in the *Firmicutes/Bacteroidetes* ratio, indicating a change in energy homeostasis during pregnancy. Similar to our findings on the *Firmicutes/Bacteroidetes* ratio in healthy pregnant women, Zheng et al. (2020) reported that there were elevations in the *Firmicutes/Bacteroidetes* ratio in the second (T2) trimester compared with the first (T1) trimester. Ley et al. (2006) reported that the *Firmicutes/Bacteroidetes* ratio decreases with weight loss on a low-calorie diet. In our observations, the *Firmicutes/Bacteroidetes* ratio did not change in GDM-W2 samples under diet management compared to the ratio in GDM samples, suggesting that the diet intervention could play a positive role during GDM pregnancy by affecting *Firmicutes/Bacteroidetes* ratio. In particular, the gut microbial pattern was not altered in the GDM group with or without 2 weeks of diet intervention (**Figures 4** **A, B**). In agreement with our observation, a controlled-feeding study showed that enterotype identity remained stable during the 10-day study, and alternative microbial states were associated with a long-term diet (Wu et al., 2011). Thus, we presume that the role of short-term diet management in GDM processes is associated with changes in the *Firmicutes/Bacteroidetes* ratio and some specific taxa rather than an alternative gut microbial pattern.

It is well suggested that the diet contributes to the gut microbiota composition in GDM (Zheng et al., 2020). Microbiota-derived metabolites affect glucose homeostasis through intestinal gluconeogenesis (De Vadder et al., 2016). A few studies have examined the gut microbiota of GDM and healthy pregnant women before and after diet invention. Uniquely, in the present study, we could compare gut microbiota in GDM fecal samples, allowing identification of taxa that exhibited differential abundance at the two time points. We discovered that a short-term diet had a beneficial effect on GDM by modulating the *Firmicutes/Bacteroidetes* ratio and some taxa. This first observation of the high prevalence of the phylum *Acidobacteria* in GDM offered an important clue for further study on the subgroup of *Acidobacteria* and the mechanism of GDM. Several limitations in our study should be considered. One was that we did not have fecal samples after long-term dietary management. Additionally, our suggestion of the occurrence of specific taxa with divergent metabolites calls for future metagenomic sequencing to reveal the metabolic pathways of the key taxa. In conclusion, our results highlight the relevance of characterizing gut microbial population differences and contribute to understanding the plausible link between diet and specific gut bacterial species that are able to influence metabolic homeostasis and GDM development. Modulating the gut microbiota *via* short-term diet intervention, especially SCFA-producing bacteria, could be a promising strategy in the search for alternatives for the treatment of metabolic disorders in GDM (Conterno et al., 2011; Clarke et al., 2014; Boulange et al., 2016). Long-term observation may be more valuable to study the dynamic alteration of the GDM gut microbiota.

## Data Availability Statement

The datasets presented in this study can be found in online repositories. The names of the repository/repositories and accession number(s) can be found below: https://ngdc.cncb.ac.cn/gsa/, CRA004782.

## Ethics Statement

The studies involving human participants were reviewed and approved by Conjoint Health Research Ethics Board of Peking University People’s Hospital. The patients/participants provided their written informed consent to participate in this study.

## Author Contributions

Sample collection and DNA extraction: JZ, ML, YY, AL, and QZ. Statistical analyses: HM, QM, HS, and JZ. Sequencing analyses and management: NW, WY, and JX. Project supervision and manuscript writing: NW, PL, and GL. All authors contributed to the article and approved the submitted version.

## Funding

This work was supported by the National Natural Science Foundation of China (grant 32070116) and the Maternal and Infant Nutrition & Care Research Fund of the Institute of Nutrition and Nursing of Biostime (grant 2015-Z-20).

## Conflict of Interest

The authors declare that the research was conducted in the absence of any commercial or financial relationships that could be construed as a potential conflict of interest.

## Publisher’s Note

All claims expressed in this article are solely those of the authors and do not necessarily represent those of their affiliated organizations, or those of the publisher, the editors and the reviewers. Any product that may be evaluated in this article, or claim that may be made by its manufacturer, is not guaranteed or endorsed by the publisher.
