# Supplementary material for: The Gut Microbial Signature of Gestational Diabetes Mellitus and the Association With Diet Intervention
Source: Front Cell Infect Microbiol. 2022 Jan 14;11:800865. doi: 10.3389/fcimb.2021.800865 (PMC8795975; doi:10.3389/fcimb.2021.800865)
Supplement: Supplementary file 1 [file DataSheet_1.docx]

Supplementary Material

# Supplementary Figures

**Supplementary Figure 1.** Alpha-diversity based on the Shannon index at the OTU level. Mann-Whitney test, GDM vs. healthy, ***P*<0.01, **P*<0.01

**Supplementary Figure 2.** Comparison of the relative abundance at the phylum level between the 27 GDM and 30 healthy individuals at the time of enrolment and study end.

**Supplementary Figure 3.** PLS-DA analysis indicated 49 distinct taxa with VIP score>1 between GDM samples and GDM-W2 samples. Mann-Whitney test, GDM vs. Healthy, ***P*<0.01, * *P*<0.01.

**Supplementary Figure 4.** The *Acidothermus, Granulicella, Bryobacter, Candidatus_Solibacter* belonging to the phylum *Acidobacteria* were evaluated in GDM and GDM-W2 samples. The 66.7% (18/27) GDM samples was showed decreased level of genus *Acidothermus* after two-week diet management. While 59.3% (16/27) GDM samples was showed decreased level of genus *Granulicella, Bryobacter, Candidatus_Solibacter* after two-week diet management.

**Supplementary Figure 5.** Functional prediction in GDM and healthy groups.

**Supplementary Table 1.** Barcode and linker primer information of 114 samples.
