# Supplementary figures and images for: The Gut Microbial Signature of Gestational Diabetes Mellitus and the Association With Diet Intervention
Source: Front Cell Infect Microbiol. 2022 Jan 14;11:800865. doi: 10.3389/fcimb.2021.800865 (PMC8795975; doi:10.3389/fcimb.2021.800865)

Fig S1

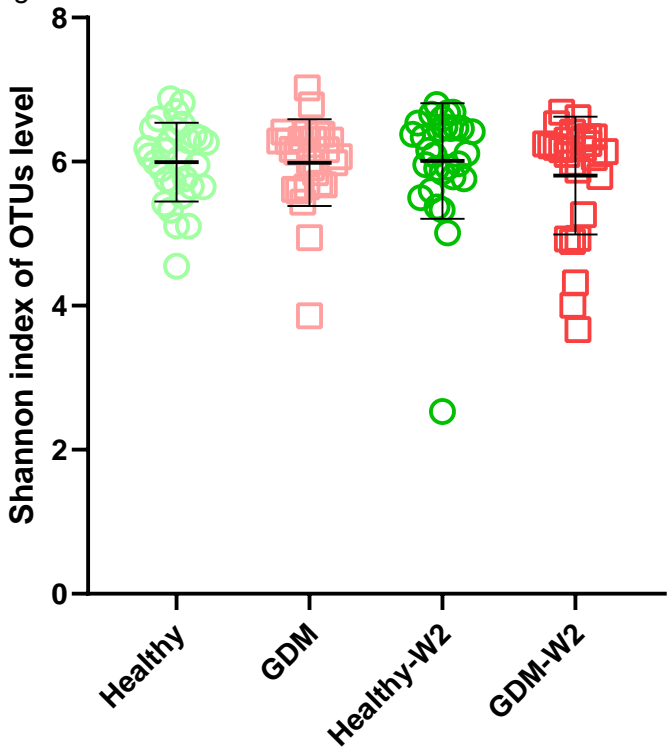

Fig S2

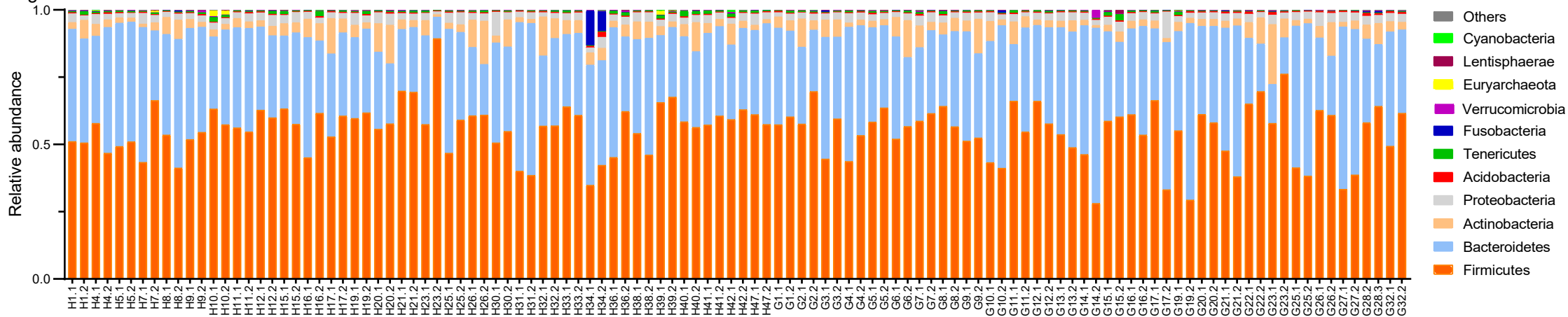

Fig S3

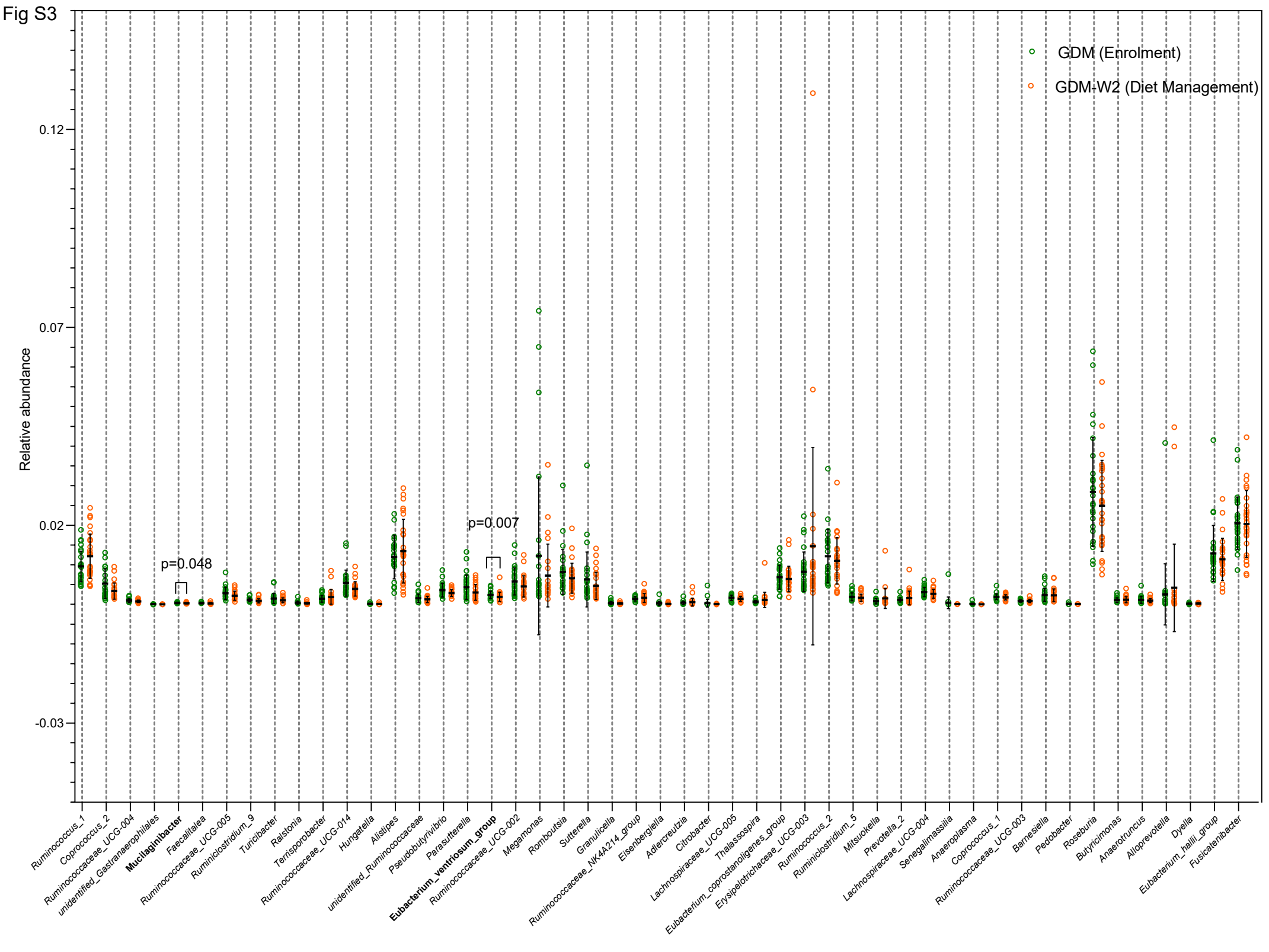

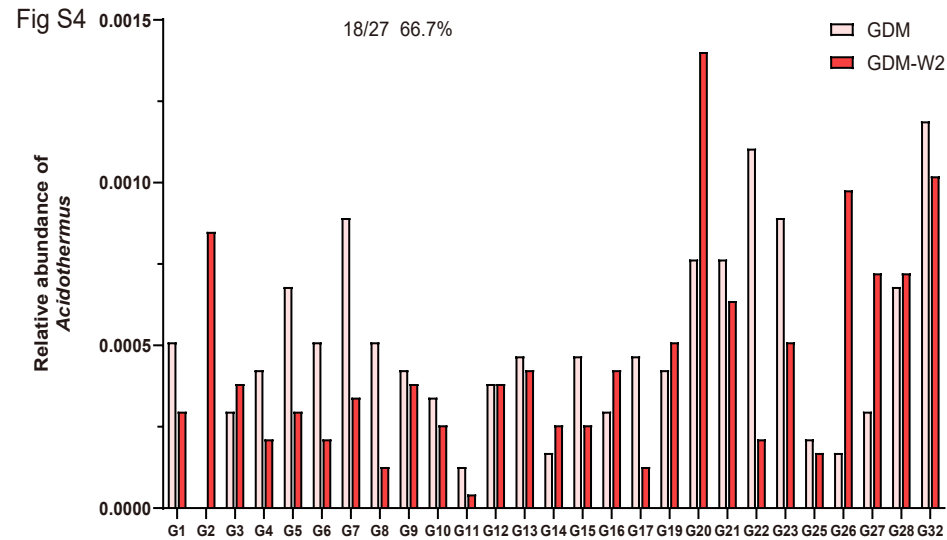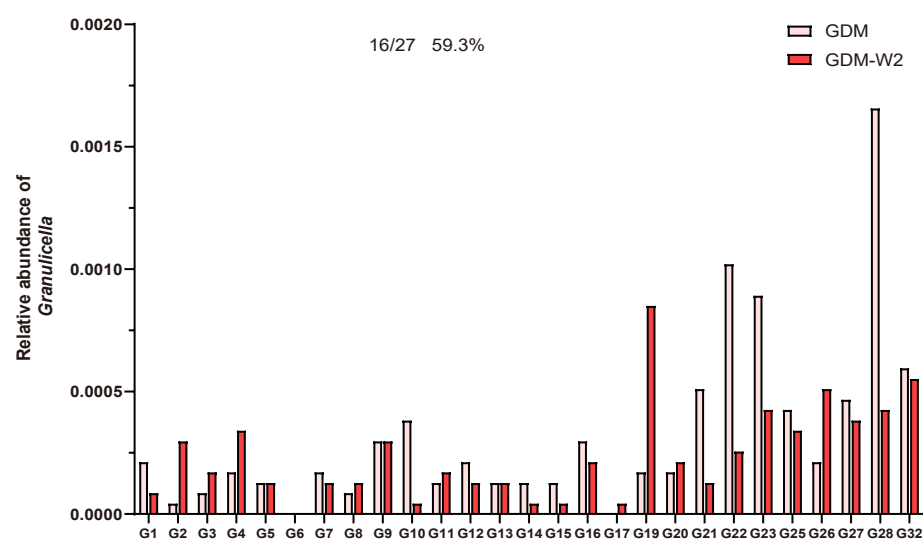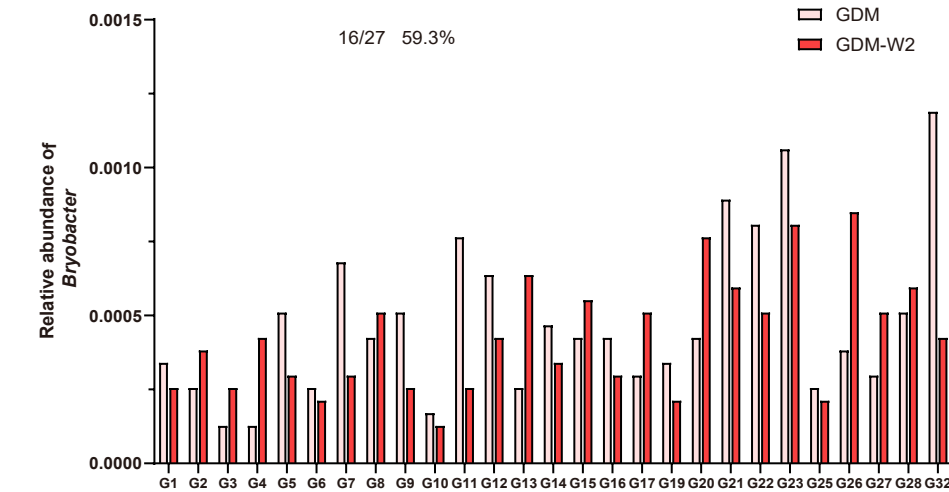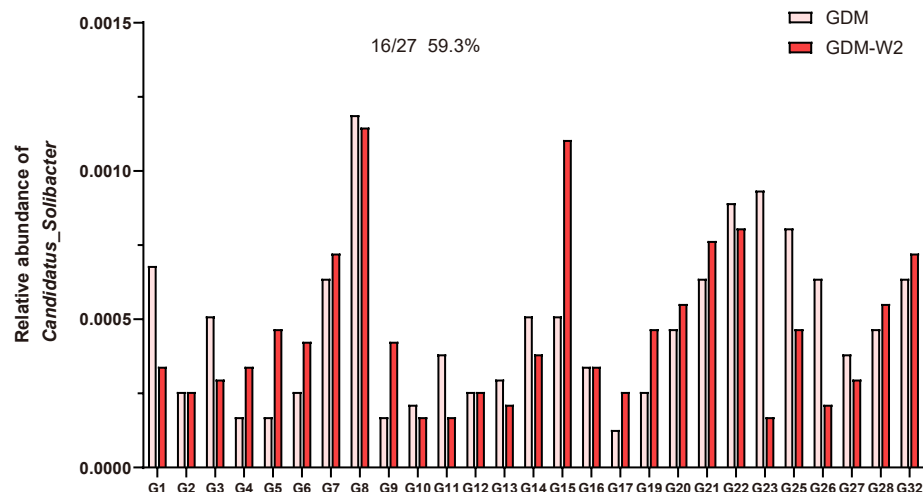

Fig S5

Healthy GDM

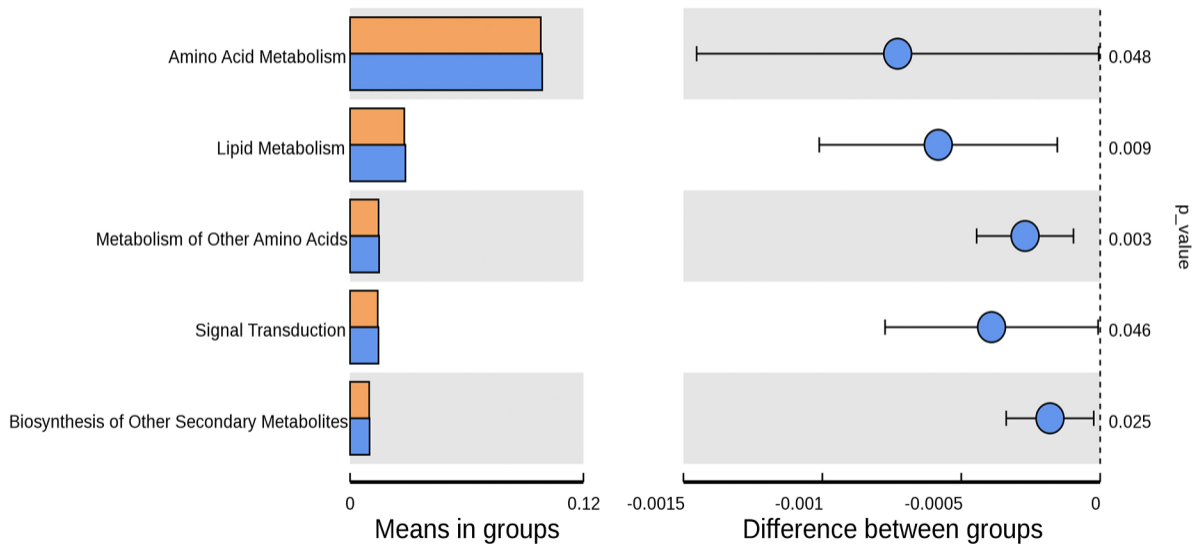

Supplement: Supplementary file 2 [file Image_1.pdf]
